# Gut-brain axis disruption, intestinal barrier damage, and systemic inflammation as predictors of POCD after cholecystectomy: a nested case-control study

**DOI:** 10.3389/fmed.2026.1833830

**Published:** 2026-06-19

**Authors:** Yanlong Fu, Qiang Wei, Huiru Chen, Xiaoyun Wu, Menghan Li, Wenxin Shi

**Affiliations:** 1 Department of Hepatobiliary Surgery, The Bethune International Peace Hospital, Shijiazhuang, Hebei, China; 2 North China University of Science and Technology, Hebei, China; 3 Department of Gastroenterology, The Bethune International Peace Hospital, Shijiazhuang, Hebei, China; 4 Department of General Smurgery, The Bethune International Peace Hospital, Shijiazhuang, Hebei, China; 5 Department of Obstetrics and Gynecology, The Second Hospital of Hebei Medical University, Shijiazhuang, Hebei, China

**Keywords:** cholecystectomy, gut-brain axis, interleukin-6, lipopolysaccharide-binding protein, neuroinflammation, postoperative cognitive dysfunction, systemic inflammation

## Abstract

**Background:**

Postoperative cognitive dysfunction (POCD) is a common complication following cholecystectomy, and the full pathogenesis remains multifactorial. Accumulating evidence suggests that the gut–brain axis plays a critical role in the development of POCD, but the specific relationship linking intestinal barrier dysfunction, systemic inflammation, and cognitive impairment has not been quantitatively characterized in cholecystectomy patients.

**Methods:**

This prospective nested case-control study enrolled 361 consecutive patients scheduled for elective cholecystectomy. Patients with POCD were identified using the Reliable Change Index (RCI). 24 POCD patients were matched 1:2 with 48 non-POCD controls. Serum levels of lipopolysaccharide-binding protein (LBP), interleukin-6 (IL-6), TNF-α, and neuronal injury markers were detected.

**Results:**

24 (6.7%) developed POCD. Baseline characteristics were well balanced. POCD patients exhibited dysregulated gut–brain axis biomarkers and a more pronounced, sustained postoperative inflammatory response. Multivariate regression analysis identified postoperative LBP (per 1 μg/mL increase; aOR = 1.08, 95%CI = 1.03–1.13, *p* = 0.001), peak postoperative IL-6 (per 1 pg/mL increase; aOR = 1.02, 95%CI = 1.01–1.03, *p* < 0.001), and peak postoperative TNF-α as independent risk factors for POCD. Higher preoperative MMSE score was a protective factor (aOR = 0.85, 95%CI = 0.73–0.99, *p* = 0.038). A greater decline in ΔMMSE was associated with an increased POCD risk.

**Conclusions:**

This study confirms a stable and independent association between intestinal leakage, systemic inflammation, and POCD following cholecystectomy. LBP and IL-6 are key biomarkers in this pathway, with high predictive value for POCD risk. The gut–brain axis drives postoperative neuroinflammation, thus supporting targeted strategies for the prevention and treatment of POCD.

## 1 Introduction

Postoperative cognitive dysfunction (POCD) refers to a postoperative neurological complication characterized by impairments in memory, attention, executive function, and information processing speed, which can lead to prolonged hospital stay, reduced quality of life, and increased long-term cognitive risk (1–6). POCD is associated with prolonged hospitalization, increased healthcare costs, reduced quality of life, and an elevated risk of long-term cognitive decline (1, 6). While elderly patients are at highest risk, POCD can affect surgical patients across different age groups (7, 8). Advances in surgical and anesthetic techniques have reduced mortality and the incidence of severe complications, making improvements in postoperative quality of life—such as cognitive function—increasingly important. Consequently, POCD has attracted increasing research attention.

Cholecystectomy is one of the most common abdominal surgeries worldwide, providing a suitable model for studying POCD (9). Although this procedure is minimally invasive, it still triggers a systemic stress response. The pathogenesis of POCD remains incompletely understood, but neuroinflammation is widely recognized as a core pathway (10). It has been proposed that the gut microbiota may modulate both preoperative and postoperative cognitive outcomes through systemic inflammation (10). Surgical tissue injury activates the innate immune system, inducing the release of proinflammatory cytokines that can compromise the blood–brain barrier and disrupt neuronal function (11–13). However, the mechanisms underlying this inflammatory response remain incompletely elucidated (14).

Bile acids are synthesized in the liver and subsequently metabolized by the gut microbiota into secondary metabolites, which serve as key regulators of microbial composition and host immune homeostasis (15). Notably, certain bile acids exert antiinflammatory effects by activating nuclear receptors such as the farnesoid X receptor (FXR) and the G protein-coupled receptor TGR5, thereby enhancing intestinal barrier integrity (16, 17). This bidirectional interaction between bile acid metabolism and the gut microbiota highlights this interaction's potential association with systemic inflammatory conditions, including POCD.

The gut–brain axis has emerged as a key pathway linking peripheral inflammation to central nervous system function (18). Surgery, anesthesia, and perioperative fasting can impair intestinal barrier integrity (intestinal barrier disruption), permitting translocation of bacterial endotoxins, including lipopolysaccharide (LPS), into the circulation (19). LBP serves as a biomarker of endotoxin exposure, thereby inducing systemic inflammation (20). It is well established that gut-derived inflammation can drive POCD; however, whether this pathway is specifically activated following cholecystectomy—and which biomarkers best capture this process—remains to be quantified in this surgical population.

Although previous studies have established the gut–brain axis as a key pathway to POCD, quantitative data on intestinal barrier dysfunction as an upstream trigger specifically after cholecystectomy are limited. The relationship between intestinal permeability markers (e.g., LBP) and elevated systemic cytokines (e.g., IL-6, TNF-α) following cholecystectomy and cognitive outcomes remains to be elucidated. This study aims to assess this gut–brain pathway in cholecystectomy patients and identify specific biomarkers (LBP, IL-6, TNF-α) associated with POCD risk.

## 2 Methods

### 2.1 Study design and patient recruitment

This was a nested case-control study derived from a prospective cohort study conducted at the Bethune International Peace Hospital, involving patients undergoing elective cholecystectomy between July 2023 and July 2024. The study protocol was approved by the Ethics Committee of the Bethune International Peace Hospital (Ethics Approval No.: 2021-KY-910), and written informed consent was obtained from all participants.

We recruited adult patients aged 60–80 years who were scheduled to undergo laparoscopic or open cholecystectomy under general anesthesia. Exclusion criteria were rigorously applied to minimize confounding factors, including: (1) a history of significant neurological or psychiatric disorders (e.g., dementia, schizophrenia, or major depressive disorder); (2) a history of severe cerebrovascular disease (e.g., stroke with significant residual functional impairment); (3) active infection or chronic systemic inflammatory disease (e.g., rheumatoid arthritis, inflammatory bowel disease) at baseline; (4) severe hepatic impairment (Child-Pugh class B or C) or renal impairment (estimated glomerular filtration rate < 30 mL/min/1.73 m^2^); (5) history of alcohol or substance abuse within the past year; and (6) any condition that precluded valid completion of the neuropsychological assessment battery (e.g., severe visual or hearing impairment, illiteracy).

Intraoperative complications were recorded during surgery; no cases of intraoperative bile leakage or intestinal injury occurred in all enrolled patients, and all operations were performed without severe surgical adverse events.

### 2.2 Neuropsychological assessment and POCD definition

A standardized battery of neuropsychological tests, administered by trained researchers blinded to patient group assignments, was used to assess cognitive function. This test battery took approximately 30 min and was conducted on the day before surgery (T0), the first day after surgery (T1), and the second day after surgery (T2). It encompassed evaluations across multiple cognitive domains: (1) memory: verbal memory tests; (2) global cognition: Mini-Mental State Examination (MMSE) or Montreal Cognitive Assessment (MoCA). POCD was defined using the RCI method, a standardized psychometric approach that accounts for practice effects and measurement variability arising from repeated cognitive assessments. Namely, POCD was defined at postoperative day 2 (T2) using the RCI method. For each cognitive test (MMSE and MoCA), an RCI score was calculated for every participant using the following formula:


$$\begin{array}{l} RCI=\frac{postoperative\;score-preoperative\;baseline\;score}{SEdiff} \end{array}$$ (1)

where SE_diff_ denoted the standard error of the difference, derived from test-retest reliability data. Given the absence of an independent healthy retest cohort, internal consistency reliability (Cronbach's α) was estimated using the baseline scores of the entire study population (*n* = 361), and SE_diff_ was computed as:


$$\begin{array}{l} SE_{\text{diff}}=S\times \sqrt{2}\times \sqrt{1-\alpha } \end{array}$$ (2)

### 2.3 Selection of cases and controls

Based on the neuropsychological assessment conducted at T2 (the second postoperative day), patients were classified into the POCD group (cases) or the non-POCD group (controls). To enhance the robustness of the comparative analysis, a 1:2 matched case-control design was implemented within the larger cohort. Each patient with POCD was individually matched with two patients without POCD based on key potential confounders: age (±5 years), sex, years of education (±3 years), and baseline Mini-Mental State Examination (MMSE) score (±2 points).

### 2.4 Blood sample collection and biomarker analysis

Peripheral venous blood samples were collected at three time points: preoperatively after induction of anesthesia (T0), and at 24 h (T1) and 48 h (T2) postoperatively. Samples were immediately centrifuged at 3,000 rpm for 15 min at 4 C. The separated serum was aliquoted and stored at −80 C for batch analysis. Serum concentrations of biomarkers related to the gut–brain axis and systemic inflammation were quantified using enzyme-linked immunosorbent assay (ELISA).

### 2.5 Enzyme-linked immunosorbent assay (ELISA)

Serum concentrations of the biomarkers were measured using commercial ELISA kits (Sigma-Aldrich for IL-6, TNF-α, S100β and BDNF; Sangon Biotech for NSE, Zonulin and CRP; Proteintech for LBP and GFAP), according to the manufacturers' instructions. All samples were assayed in duplicate to ensure reproducibility. The optical density was read at 450 nm using a microplate reader, and the concentrations were calculated from the standard curves.

### 2.6 Statistical analysis

Statistical analyses were performed using SPSS (version 25.0). The Shapiro-Wilk test was applied to assess the normality of all continuous variables. Normally distributed continuous data were presented as mean ± standard deviation (SD), while non-normally distributed data were described as median (interquartile range, IQR). Categorical data were expressed as *n* (%). Baseline characteristics between groups were compared using the Student's t-test for normally distributed continuous variables or the Mann-Whitney U test for non-normal variables. The χ^2^ test or Fisher's exact test was adopted for categorical comparisons as appropriate. Differences in longitudinal biomarker levels were analyzed using two-way repeated-measures ANOVA for normally distributed data or the Friedman test for non-normal data. Linear regression analysis was performed to evaluate the association between changes in MMSE scores (ΔMMSE) and POCD risk. A two-tailed *p* < 0.05 was considered statistically significant.

## 3 Result

### 3.1 Cohort derivation and baseline equipoise

The present analysis was derived from a prospective observational cohort of 361 consecutive patients scheduled for elective cholecystectomy. Application of the RCI to postoperative neuropsychological testing identified 24 individuals with a diagnosis of POCD. Using a 1:2 nested case-control design, each case was matched with two controls from the n-POCD pool based on age, sex, and preoperative cognitive score, yielding a final analytical sample of 72 subjects. Demographic and clinical profiles for these matched cohorts were compiled in Table 1. Critically, the matching protocol effectively balanced the groups, as evidenced by the absence of significant disparities in age, body mass index, educational attainment, or the prevalence of comorbid conditions such as hypertension and diabetes mellitus (p>0.05). This successful matching mitigated concerns that pre-existing patient factors could confound the observed biological and cognitive outcomes.

**Table 1:** Comparison of baseline demographic and clinical characteristics among the two groups.

Variable			POCD (*n* = 24)	N-POCD (*n* = 48)	*P*
Demographic information	Age (year)^a^		67.16 ± 10.2	70.23 ± 9.8	0.123
	Gender (M.%)^b^		11 (45.8%)	26 (54.2%)	0.249^Δ^
	Education (years)		8.18 ± 8.1	7.16 ± 4.2	0.218
	BMI (kg/m^2^)		28.9 (24.1, 35.8)	30.1 (24.7, 36.8)	0.535
	ASA status *n* (%)	I	13(54.2%)	21(43.7%)	0.057
		II	11 (45.8%)	27 (56.3%)	0.075
Preoperative cognitive score	MMSE score		27.1 ± 1.6	27.6 ± 1.9	0.441
	MOCA score		26 (24, 28)	26 (23, 27)	0.153
Surgery and anesthesia	Duration of the operation (min)^c^		91.5 ± 26.4	89.5 ± 28.9	0.145
	Duration of anesthesia (min)^c^		125.5 (135.5, 170.0)	138.5 (133.0, 167.5)	0.345
	Intraoperative blood loss (ml)^c^		10 (5, 15)	15 (5, 20)	0.289
Postoperative condition	Postoperative complication (%)		6 (25.0%)	13 (27.1%)	0.545
	Postoperative hospital stay (days)^a^		4.6 ± 1.4	4.7 ± 0.8	0.356

Values were presented as mean ± standard deviation or number (percentage).

M, male; F, female; BMI, body mass index; MMSE, mini-mental state examination; MoCA, Montreal cognitive assessment; ASA, American society of anesthesiologists; SD, standard deviation; IQR, interquartile range.

^a^data presented as mean ± SD.

^b^data presented as *n* (%).

^c^data presented as median (IQR).

Δ*P* value: Chi-square test. × *P* value: independent t-test.

### 3.2 Distinct biomarker profiles at baseline and following surgery

**Figure 1:**
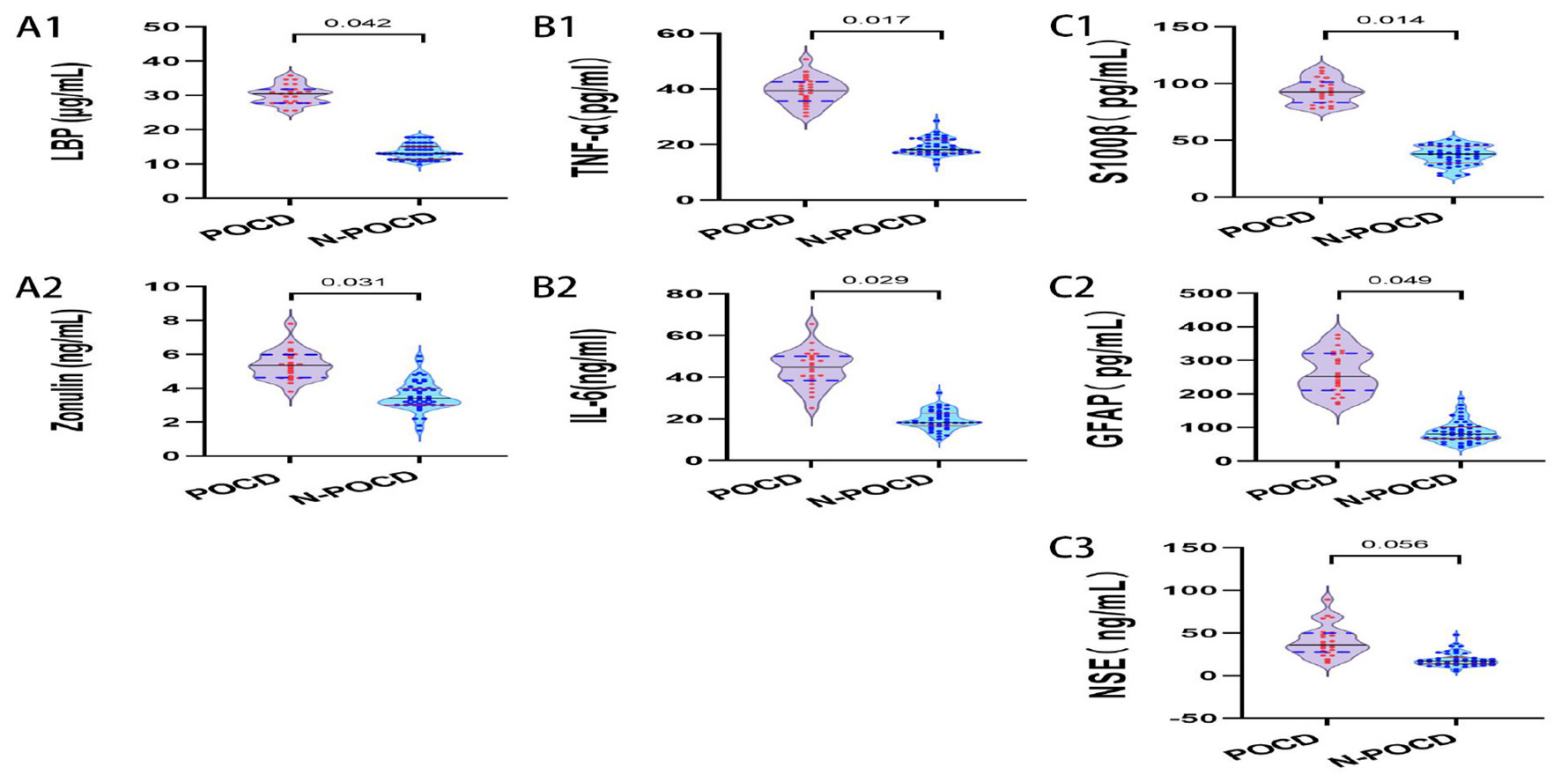
Comparison of gut–brain axis biomarker levels between the POCD and n-POCD groups. Box plots depict serum concentrations of **(A)** gut permeability markers (LBP and Zonulin), **(B)** pro-inflammatory cytokines (IL-6 and TNF-α), and **(C)** neuronal injury markers (GFAP, S100β, and NSE) in patients who developed POCD (*n* = 24) and their matched controls (*n* = 48). *P* < 0.05 for all comparisons between the POCD and n-POCD groups across all biomarkers except NSE.

POCD patients exhibited dysregulation of biomarkers across the gut–brain axis. As shown in Figure 1, significant differences were observed not only in intestinal permeability markers (LBP and Zonulin) but also across a range of systemic inflammatory cytokines and neuronal injury markers. Specifically, the proinflammatory cytokines—including IL-6 and TNF-α–were significantly elevated in the POCD group. For instance, median levels of IL-6 and TNF-α were higher in this group than in the control group (*p* < 0.05). These findings indicate a state of systemic inflammation. Furthermore, a concomitant rise in biomarkers indicative of early neuroglial activation and injury was observed. Serum levels of the astroglial markers glial fibrillary acidic protein (GFAP) and S100 calcium-binding protein B (S100β) were significantly elevated in the POCD group. A similar upward trend was also observed for neuron-specific enolase (NSE).

### 3.3 Dysregulation across the gut-brain axis spectrum

**Figure 2:**
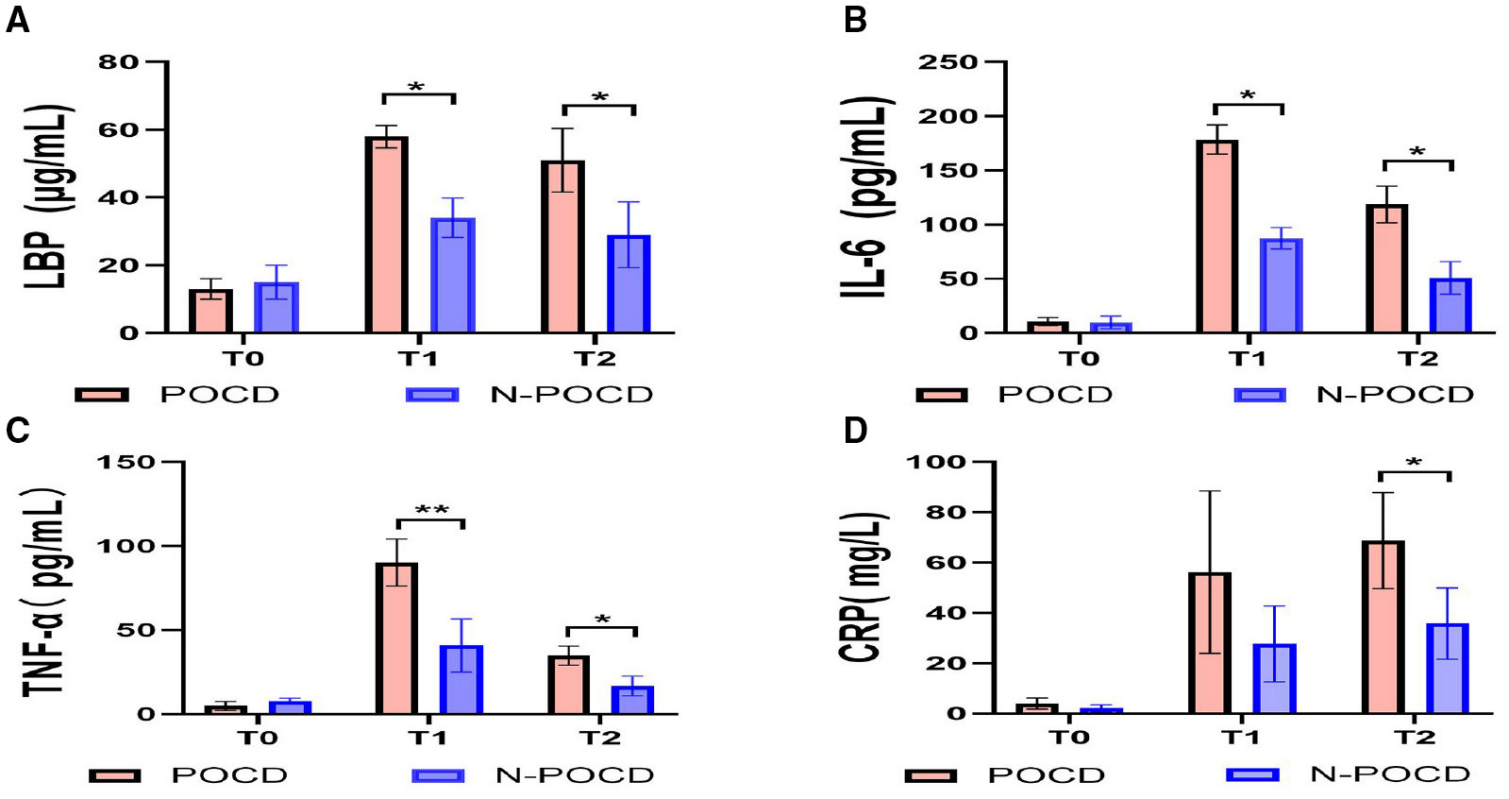
Perioperative kinetics of gut permeability and systemic inflammatory markers in patients with and without POCD. This illustrates the longitudinal changes in serum concentrations of key biomarkers at T0, T1 and T2 in patients with and without POCD. **(A)** LBP; **(B)** IL-6; **(C)** TNF-α; and **(D)** CRP. The biological indicators of both groups increased on T1; however, the POCD group exhibited a significantly more pronounced and sustained inflammatory response. This exaggerated reaction was paralleled by persistently elevated LBP levels in the POCD group.*p < 0.05, **p < 0.01, ***p < 0.001 indicate significant differences between groups at the corresponding time points.

Despite comparable baseline demographics, the cohorts exhibited pronounced differences in a panel of biomarkers reflecting intestinal barrier integrity and systemic inflammation. Serum concentrations of LBP and key proinflammatory cytokines, including IL-6 and TNF-α, were elevated in patients who developed POCD. The POCD group exhibited a distinct and sustained inflammatory response (Figure 2). Although both groups exhibited elevations in IL-6, TNF-α, and C-reactive protein (CRP) on postoperative days 1 and 2, these increases were significantly more pronounced in patients who developed POCD. The parallel rise in LBP alongside these cytokine shifts further suggests a potential gut-derived component driving this sustained systemic inflammatory state.

### 3.4 Identification of independent pre- and perioperative risk factors for POCD

Results of multivariate regression analysis showed that elevated levels of markers associated with intestinal barrier function and systemic inflammation were significantly and independently associated with the risk of POCD (Table 2).

**Table 2:** Multivariable logistic regression analysis of independent risk factors for POCD.

Variable	Univariable analysis		Multivariable analysis	
Univariable analysis	OR (95% CI)	*p*-value	aOR (95% CI)	*p*-value
Postoperative LBP (per 1 μg/mL increase)	1.15 (1.09–1.21)	< 0.001	1.08 (1.03–1.13)	0.001
Postoperative IL-6 (per 1 pg/mL increase)	1.04 (1.03–1.05)	< 0.001	1.02 (1.01–1.03)	< 0.001
Age (per 5-year increase)	1.60 (1.30–1.97)	< 0.001	1.45 (1.15–1.83)	0.002
Preoperative MMSE score (per 1-point increase)	0.80 (0.70–0.92)	0.001	0.85 (0.73–0.99)	0.038

OR, odds ratio; aOR, adjusted odds ratio; CI, confidence interval.

Specifically, postoperative serum LBP levels (per 1 μg/mL increase) were an independent risk factor for POCD [adjusted odds ratio (aOR) = 1.08, 95% confidence interval (CI) = 1.03–1.13, *p* = 0.001], suggesting that intestinal barrier disruption may play a role in the pathogenesis of POCD. Furthermore, the intensity of the postoperative systemic inflammatory response was also independently associated with POCD; specifically, the peak level of postoperative IL-6 (per 1 pg/mL increase; aOR = 1.02, 95% CI = 1.01–1.03, *p* < 0.001) and the peak level of TNF-α were both confirmed as independent risk factors for POCD. On the other hand, univariate analysis showed that a higher preoperative MMSE score (per 1-point increase) was associated with a reduced risk of POCD [odds ratio (OR) = 0.80, 95% CI = 0.70–0.92, *p* = 0.001]. After further adjustment for potential confounding factors such as age, sex, and comorbidities, this protective effect persisted; although it was slightly weakened, it remained statistically significant (aOR = 0.85, 95% CI = 0.73–0.99, *p* = 0.038).

### 3.5 Correlation between perioperative biomarker dynamics and cognitive decline

**Figure 3:**
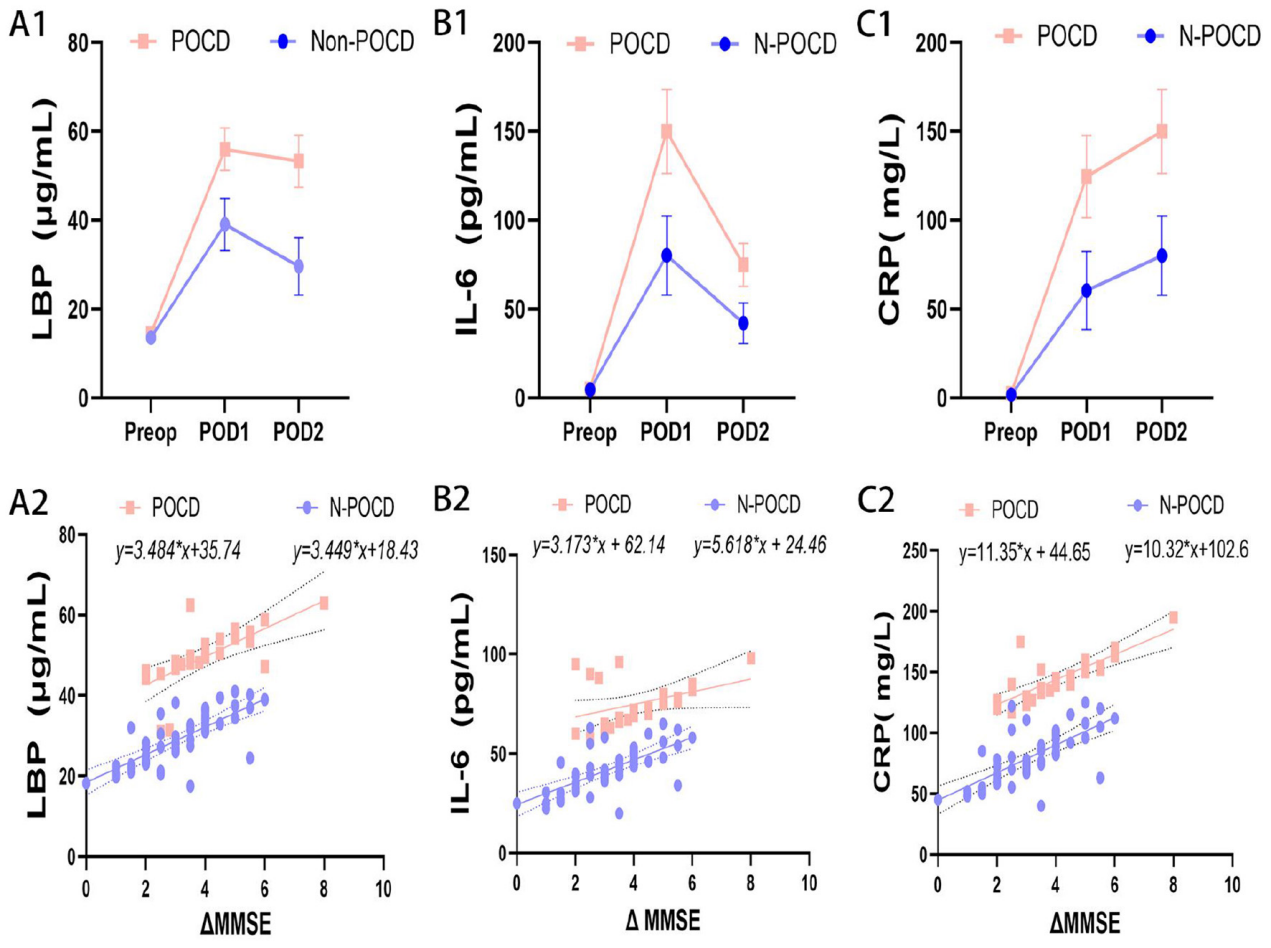
Dynamic changes of MMSE scores and their correlation with postoperative cognitive dysfunction (POCD). A1–A2: MMSE score changes from preoperative (Preop) to postoperative day 1 (POD1) and POD2 in n-POCD **(A1)** and POCD **(A2)** groups; B1–B2: Correlation between ΔMMSE (changes in MMSE scores) and cognitive function in n-POCD **(B1)** and POCD **(B2)** groups; C1–C2: Linear regression fitting curves of ΔMMSE and cognitive function in n-POCD **(C1)** and POCD **(C2)** groups. Linear regression was used for correlation analysis. Abbreviations: ΔMMSE, Changes in MMSE scores; Preop, Preoperative; POD1, Postoperative day 1; POD2, Postoperative day 2.

Dynamic changes in MMSE scores and their correlation with POCD were further analyzed, and the results are shown in Figure 3. Specifically, the MMSE scores of both the POCD group and the non-POCD group showed distinct trends from preoperative (Preop) to postoperative day 1 (POD1) and postoperative day 2 (POD2).

In the non-POCD group, the MMSE score showed a relatively stable trend during the perioperative period, with a mild fluctuation but no significant decline (Figures 3A1, B1, C1). In contrast, the POCD group exhibited a notable decrease in MMSE scores after surgery, with the lowest level observed on POD1 or POD2, indicating impaired cognitive function in patients with POCD (Figures 3A2, B2, C2).

Linear regression analysis revealed a significant correlation between ΔMMSE (changes in MMSE scores, ΔMMSE) and the risk of POCD. The fitting equations showed that ΔMMSE was positively associated with cognitive function, suggesting that a greater decrease in ΔMMSE was closely related to the occurrence of POCD.

## 4 Discussion

This study provides compelling clinical evidence further supporting the established role of the gut–brain axis in the pathogenesis of POCD following cholecystectomy. By combining a matched cohort design with robust biomarker analysis, we quantitatively characterized a distinct pathway from surgical injury to cognitive decline. Our primary findings consistently demonstrate that: (1) Patients who developed POCD exhibited markedly stronger postoperative inflammatory responses, with elevated levels of both gut-derived (LBP) and systemic (IL-6, TNF-α) inflammatory markers compared to matched controls (Figure 1); (2) The dynamic trajectory of this inflammatory surge was significantly steeper in POCD patients from the preoperative to postoperative period (Figure 2); (3) Postoperative LBP and IL-6 levels emerged as independent risk factors for POCD after rigorous adjustment for confounders (Table 2); and (4) The intensity of this inflammatory response directly correlated with the severity of cognitive deterioration and exhibited significant predictive power for identifying high-risk individuals (Figure 3). Collectively, these data robustly support the framework that cholecystectomy can induce intestinal barrier dysfunction, triggering a systemic inflammatory cascade that ultimately disrupts brain function and manifests as POCD.

Building on these findings, the most salient contribution of our work is the elucidation of gut-derived inflammation as a critical factor in POCD. The robust and independent association between postoperative LBP and POCD forms the cornerstone of this finding. LBP is an acute-phase protein synthesized in response to circulating LPS, the primary component of the Gram-negative bacterial outer membrane (21–23). Elevated systemic LBP levels thus serve as a sensitive and specific surrogate marker for the translocation of bacterial products from the gut lumen into the systemic circulation—a phenomenon indicative of increased intestinal permeability, or intestinal barrier disruption (24). Notably, the synchronized and parallel elevations of LBP, IL-6, and TNF-α observed in the POCD group construct a compelling pathophysiological narrative linking intestinal barrier disruption to systemic inflammation and subsequent cognitive impairment. Abdominal surgery, including minimally invasive procedures such as laparoscopic cholecystectomy, is known to induce physiological stress, splanchnic hypoperfusion, and neurohormonal changes—all of which compromise the integrity of the intestinal epithelial barrier (25–27). This breach allows the egress of LPS into the portal circulation and subsequently into the systemic circulation. Upon binding to pattern recognition receptors—such as Toll-like receptor 4 (TLR4)—on immune cells, LPS initiates a potent signaling cascade that culminates in the massive release of proinflammatory cytokines, including IL-6 and TNF-α (28–30). Our data indicate that the POCD group exhibited a significantly greater inflammatory surge from comparable baseline levels (Figure 2). This suggests that these patients may have a heightened vulnerability to this gut-initiated inflammatory pathway, potentially due to more severe intestinal barrier disruption or an exaggerated immune response.

Collectively, these observations provide clinical validation for the emerging model of POCD pathogenesis that positions the gastrointestinal tract not as a passive organ, but as a key effector and amplifier of the systemic inflammatory response to surgery. This aligns with and quantifies the concept that the gut acts as a central hub that propagates and sustains systemic inflammatory states, offering a coherent explanation—grounded in specific biomarkers (LBP, IL-6)—for how a peripheral event can lead to central nervous system (CNS) dysfunction.

The identification of IL-6 as a powerful independent predictor of POCD (Table 2) solidifies the link between peripheral inflammation and cerebral consequences. IL-6 is a multifunctional cytokine playing a central role in the acute-phase response. While essential for host defense, its sustained elevation is pathogenic (31–34). Our findings align with extensive literature linking IL-6 to delirium and cognitive decline across diverse clinical settings—from cardiopulmonary bypass surgery to critical illness, including intensive care units and emergency departments (35, 36). Researchers are investigating how these peripheral cytokines communicate with the CNS. Several mechanisms are plausible and not mutually exclusive. Circulating cytokines such as IL-6 and TNF-α can actively cross the damaged blood–brain barrier (BBB) *via* saturated transport systems (37). TNF-α has been demonstrated to synergize with IL-6 in promoting the migration of autoimmune lymphocytes and monocytes into the CNS (38). IL-6 and TNF-α disrupt tight junctions, leading to the breakdown of the endothelial barrier (39). The interactions between these cytokines are particularly crucial in autoimmune neuroinflammatory diseases. In these conditions, the sustained activation of IL-6 and TNF-α continuously triggers inflammatory responses and BBB leakage, ultimately leading to neuronal damage and cognitive impairment (40).

Notably, IL-6 and TNF-α are acute inflammatory markers that inevitably rise postoperatively due to surgical stress. However, baseline characteristics and surgical trauma were well balanced between groups, minimizing nonspecific inflammatory confounding. Furthermore, LBP is a relatively specific marker of intestinal barrier disruption rather than a generic acute-phase reactant. After adjustment for perioperative confounders, LBP, IL-6, and TNF-α remained independently associated with POCD, indicating that their elevations reflect gut-derived inflammatory activity relevant to POCD pathogenesis rather than nonspecific surgical stress alone.

The observed correlation between serum inflammation levels and the degree of cognitive decline (Figure 3) provides compelling indirect evidence that peripheral inflammation influences brain injury. This translation likely occurs through one or more pathways. Neuroinflammation, characterized by altered neuronal cell function (41), leads to synaptic dysfunction, oxidative stress, and ultimately disrupts neural networks in postoperative cognitive impairment (42).

Our findings deepen the current understanding of POCD. Numerous studies have established associations between common inflammatory markers (e.g., CRP, IL-6) and POCD (43, 44). However, many studies have posited that systemic inflammation originates from surgical site injury. Our study provides clinical evidence supporting the concept that postoperative inflammation can originate from the intestine—a distinct, potent, and treatable source—in the setting of cholecystectomy.

Multivariate regression results confirm that intestinal barrier dysfunction and systemic inflammation are independently associated with POCD risk. Postoperative LBP, a surrogate for intestinal permeability, supports intestinal barrier disruption as a contributor to POCD pathogenesis. Postoperative peak IL-6 and TNF-α also independently predict POCD, highlighting the role of sustained systemic inflammation in neuroinflammation and cognitive impairment. Higher preoperative MMSE scores persistently reduce POCD risk, indicating baseline cognitive reserve as a potential preoperative risk stratification target. These findings underscore the gut–brain axis in POCD and the clinical value of LBP, IL-6, and preoperative MMSE for risk assessment. These findings align with emerging evidence implicating gut-derived toxins as key contributors to brain dysfunction in conditions such as sepsis-associated encephalopathy and hepatic encephalopathy (45, 46).

The novelty of our study lies in applying this well-established concept of the gut–brain axis to the clinical setting of elective cholecystectomy, thereby greatly expanding its potential applicability. Our findings hold dual significance: both prognostic and therapeutic. From a prognostic perspective, the combined use of LBP and IL-6 measurements in the early postoperative period can serve as a clinical point-of-care tool for POCD risk stratification. Identifying high-risk patients upon admission to the ward or the recovery room enables targeted monitoring, early mobilization, and non-pharmacological interventions known to benefit cognitive outcomes.

From a therapeutic perspective, given the established role of the gut–brain axis supports the rationale for targeting this pathway for preventing POCD. If increased intestinal permeability serves as a critical initiating factor in the pathological process of POCD, then enhancing intestinal barrier function may represent an effective strategy for mitigating postoperative cognitive impairment. These could include: (i) Drug formulations, such as larazotide or other zonulin inhibitors, currently being studied for the treatment of celiac disease (47–49); (ii) Nutritional support, such as fiber, arginine, and n-3 polyunsaturated fatty acids, which have been shown to help reduce infections and maintain intestinal homeostasis (46, 50–52); (iii) Microbiota-based therapies, such as strains of lactic acid bacteria or prebiotics, which aim to promote the formation of a healthy gut microbiota and enhance barrier function (53, 54).

Although our study provides strong evidence of association, several limitations must be acknowledged. First, while this observational and nested case-control design helps establish associations, it cannot prove causation. Second, the patient sample in this study was recruited from a single tertiary center, which may limit the generalizability of our findings; validation in a diverse, multicenter, and prospective cohort is essential. Third, this study assessed neuroinflammation solely through peripheral biomarkers; future research should incorporate neuroimaging or cerebrospinal fluid analysis to provide direct evidence of CNS inflammation. Fourth, this study necessitates long-term follow-up to evaluate the persistence of cognitive impairment and the long-term impact of intestinal barrier dysfunction. In addition, frailty assessment was not performed in this study. Future studies should include frailty evaluation to further improve risk stratification for postoperative cognitive dysfunction.

## 5 Conclusion

In conclusion, this study confirms a stable and independent association between intestinal leakage, systemic inflammation, and POCD following cholecystectomy. We have identified LBP and IL-6 as key biomarkers in this pathway, demonstrating their value in predicting POCD risk n patients undergoing laparoscopic cholecystectomy. Our findings further support and refine the existing pathophysiological model of POCD by providing quantitative evidence from a cholecystectomy cohort that the gut–brain axis acts as a key driver of postoperative neuroinflammation. Based on this pathway, our research identifies specific biomarkers that open promising avenues for targeted prevention and treatment of POCD.

## Data Availability

The original contributions presented in the study are included in the article/supplementary material, further inquiries can be directed to the corresponding author.
